# Adherence to key domains in low back pain guidelines: A cross‐sectional study of Danish physiotherapists

**DOI:** 10.1002/pri.1858

**Published:** 2020-06-15

**Authors:** Maja Husted, Camilla B. Rossen, Tue S. Jensen, Lone R. Mikkelsen, Nanna Rolving

**Affiliations:** ^1^ Interdisciplinary Research Unit, Elective Surgery Centre Silkeborg Regional Hospital Silkeborg Denmark; ^2^ Diagnostic Centre, University Research Clinic for Innovative Patient Pathways Silkeborg Regional Hospital Silkeborg Denmark; ^3^ Department of Sports Science and Clinical Biomechanics SDU Odense Denmark; ^4^ Department of Clinical Medicine Aarhus University Aarhus Denmark; ^5^ Public Health & Rehabilitation Research DEFACTUM Aarhus Denmark

**Keywords:** evidence‐based practice, guideline adherence, low back pain, physiotherapist

## Abstract

**Objective:**

The aim of the study was threefold: Firstly, to investigate the adherence to clinical practice guidelines for low back pain (LBP) among Danish physiotherapists with regard to three key domains: (a) activity, (b) work and (c) psychosocial risk factors. Secondly, to investigate whether adherence differed between physiotherapists working in private clinics (private physiotherapists) and physiotherapists working at public healthcare centres (public physiotherapists). Thirdly, to describe the physiotherapists' treatment modalities for patients with LBP.

**Methods:**

A cross‐sectional online survey was conducted with 817 physiotherapists working in the Central Denmark Region. Adherence to the guideline domains was assessed using two vignettes. The difference in adherence between the groups was assessed using the Chi‐squared test. Treatment modalities were reported using descriptive statistics.

**Results:**

A total of 234 physiotherapists responded, hereof 163 private physiotherapists and 71 public physiotherapists (response rate 29%). The proportions of physiotherapists managing the patients strictly in line with the guideline domains were 32% (activity), 16% (work) and 82% (psychosocial risk factors) for Vignette 1 and 6% (activity), 53% (work) and 60% (psychosocial risk factors) for Vignette 2. Public physiotherapists were more likely to manage patients strictly in line with guidelines for assessing the psychosocial risk factors compared to private physiotherapist (Vignette 1: 92% vs. 77% *p* = .030; Vignette 2:70% vs. 55% *p* = .035). Regarding the other two domains, there was no significant difference between the two groups in terms of adherence (*p* > .05). Concerning treatment modalities, the majority of physiotherapists instructed the patients in adopting an exercise program or informed the patients about the benign nature and prognosis of LBP.

**Conclusion:**

Overall, the participating Danish physiotherapists strictly adhered to only one out of three key domains. This underlines the importance of bringing focus on implementing the current guidelines' recommendations in clinical practice.

## 1 INTRODUCTION

Low back pain (LBP) is of great importance, as it is the world's leading cause of years lived with disability (Hoy et al., 2014; James et al., 2018). In Denmark, patients with LBP comprise 30% of all visits to the physiotherapist (PT) or chiropractor and 20% of all sick leave days is caused by LBP (Danish Health Authority, 2015b).

Although Clinical Practice Guidelines (CPGs) for LBP have existed in Denmark since 2012, patients continue to report incoherent care pathways with wide variation in the information and treatment they receive (Danish Health Authority, 2015a). This poses a problem, as studies have shown that adherence to CPGs can reduce inappropriate variations in the healthcare system and optimize the use of evidence‐based management. Moreover, CPG adherences is more cost‐effective, due to a reduction in the number of consultations and less disability (Fritz, Cleland, & Brennan, 2007; Grimshaw, Eccles, & Russell, 1995; Hanney, Masaracchio, Liu, & Kolber, 2016; G. M. Rutten et al., 2010). Nevertheless, a wide variation in CPG adherence and treatment modalities in clinical practice has been documented repeatedly (de Souza, Ladeira, & Costa, 2017; Hendrick, Mani, Bishop, Milosavljevic, & Schneiders, 2013; Keating et al., 2016; Ladeira, Samuel Cheng, & Hill, 2015).

Since the first CPG for LBP management was described by the Quebeck Task Force in 1987 (Spitzer, Le Blanc, & Dupuis, 1987), a number of CPGs have been published internationally (Koes et al., 2010; Oliveira et al., 2018; Pillastrini et al., 2012) and nationally in Denmark (Danish Health Authority, 2013, 2016b, 2016a, 2017). Across these CPGs, there is agreement that evidence‐based care for patients with LBP (in the absence of severe spinal pathology or other serious disease) should include information about the benign nature and prognosis of LBP, advice to stay physically active (despite pain), and advice to stay at work or return to work as soon as possible (despite pain) (Danish Health Authority, 2016a, 2016b; Koes et al., 2010; Oliveira et al., 2018; Pillastrini et al., 2012). In addition, CPGs agree that certain psychosocial factors entail a risk of developing a chronic condition and therefore should be identified and addressed to succeed in LBP management (Danish Health Authority, 2013; Koes et al., 2010; Lin et al., 2020; Oliveira et al., 2018; Pillastrini et al., 2012).

In a Danish context, management of LBP by PTs has been investigated in only one study, published in 2003 (Hamm et al., 2003). The study found that PTs often used at least one treatment recommendation according to the Health Technology Assessment report ‘Low Back Pain’ (Danish Centre for Evaluation and Health Technology Assessment., 1999), but in addition, used passive treatments such as massage, heat and cold treatment and electrotherapy, which were recommended against by the report (Hamm et al., 2003). CPGs have been published in the field of LBP management since then, but no recent studies have been conducted investigating the current level of CPG adherence and treatment modalities used by Danish PTs.

The aim of the study was threefold: Firstly, to investigate the adherence to CPGs for LBP among Danish PTs with regard to three key guideline domains: (a) activity, (b) work and (c) psychosocial risk factors. Secondly, to investigate whether adherence differed between PTs working in private clinics and PTs working at public healthcare centres. Thirdly, to describe the PTs' treatment modalities for patients with LBP.

## 2 METHODS

### 2.1 Setting

In Denmark, healthcare services are primarily free of charge and independent of the individual's income. However, in terms of private physiotherapy clinics, the public health insurance covers only 50% of the expenses. In comparison, LBP rehabilitation at public healthcare centres in the municipalities is completely free of charge. Patients at public healthcare centres can only be referred following a LBP consultation by a medical specialist in the secondary sector, whereas patients at private clinics can be referred either from their general practitioner or from the secondary sector. PTs working in public healthcare centres generally manage patients late in the care pathway following diagnosing by a medical specialist. In contrast, PTs working in private clinics treat patients in acute, subacute and chronic stages. Patients visiting private and public PTs may thus have different needs in terms of LBP management, as patients in acute and subacute stages might need more manual treatment compared to patients in chronic stages. However, regarding the three key domains the management of patients should not differ between private or public PTs.

### 2.2 Design and study population

The study had a cross‐sectional design. An online survey was conducted between February and April 2018. The eligibility criteria of the study were all PTs working in private clinics (hereafter referred to as private PTs) within health insurance and at public healthcare centres (hereafter referred to as public PTs) in the Central Denmark Region, treating at least one patient with LBP per week. The region includes 19 municipalities with approximately 1.3 million citizens in total. The private clinics and public health care centres invited to participate were identified by Sundhed.dk (Danish Regions, Ministry of Health, KL—Goverment of Health, n.d.), which is Denmark's public health website.

### 2.3 Data collection

The data were collected via an electronic survey sent by email to all private clinics working within health insurance and public healthcare centres in the Central Denmark Region. The survey was designed using Research Electronic Data Capture (REDCap) hosted at the Department of Clinical Medicine at Aarhus University (Harris et al., 2009). The email consisted of an invitation with information about the study and a link to access the electronic survey. The emails were addressed to either the managers of the public healthcare centres or the secretaries or owners of the private clinics, who were then asked to forward the email to all eligible PTs at each location. Electronic reminders and telephone calls were made after 1, 3 and 6 weeks.

### 2.4 Survey instrument

The questionnaire was designed specifically for the current study and was developed and pilot‐tested in the autumn of 2017, in collaboration with researchers and clinicians at the Regional Spine Clinic, located at Silkeborg Regional Hospital, as well as with private and public PTs.

The first section of the survey included a series of questions designed to describe the demographic characteristics of the participating PTs (e.g., age, sex and years of practice experience). The last section of the survey included two vignettes, described below.

### 2.5 Vignettes

The vignettes are written as hypothetical patient scenarios, which are considered valid instruments for measuring the clinicians' adherence to CPGs (G. M. J. Rutten, Harting, Rutten, Bekkering, & Kremers, 2006), and have previously been used in similar studies (Derghazarian & Simmonds, 2011; Hendrick et al., 2013; Keating et al., 2016; Learman, Ellis, Goode, Showalter, & Cook, 2014). In this study, all PTs were presented with two vignettes (referred to as Vignette 1 and Vignette 2). The descriptions of both vignettes were developed for this study based on real patients and were subsequently validated by five clinicians with extensive expertise in the field. Vignette 1 was designed to reflect a patient with psychosocial risk factors and reduced activity level. It represented a 35‐year‐old woman with severe LBP during the previous 12–13 weeks. She had been on sick leave since the LBP started. The MRI scan showed age‐related degenerative changes. Vignette 2 represented a 23‐year‐old man, with no sign of psychosocial or work/activity related risk factors. He had LBP with radiculopathy to the right leg for approximately 3 months. The MRI scan showed a L5‐S1 disc prolapse with signs of nerve root pressure. Neither vignettes reported red flags (signs of severe spinal pathology or other serious disease). The two vignettes are described in detail in Table 1.

**TABLE 1:** Description of the two vignettes

*Patient 1*
A 35‐year‐old woman is referred from the secondary sector following an episode of severe LBP, which has lasted for 12–13 weeks. She is married and has a 4‐year‐old child
In the past few years, she has not had the energy to be physically active. She has been on sick leave from her job as a healthcare assistant since the episode started
This is the third and worst episode of LBP she has experienced. In the two previous episodes, the pain has resolved spontaneously. The pain is currently reduced to approximately 50% of its worst intensity during this episode. The pain does not disturb her sleep. She is currently taking paracetamol and NSAID when needed
She is very concerned about the intensity of the pain and she is nervous that her back problems will not resolve this time. The patient feels she still needs to rest her back once in a while. She is afraid of exacerbating the pain again, in case she has to lift something from an awkward position
Extension and lateral flexion is moderately reduced, while flexion is limited only minimally. The neurological examination is normal. The patient experiences some sensory disturbances in the lower right leg, but the medical examination shows normal reflexes and no strength reduction. The MRI scan shows age‐related degenerative changes
*Patient 2*
A 23‐year‐old man is referred from the secondary sector following an episode of LBP with radiculopathy to the right leg. The pain started about 3 months ago without prior trauma. Various physiotherapeutic treatments have been attempted without considerable effect, for example, McKenzie exercises and manipulation
The patient has paused football and other sports activities, but has resumed running again. He still cannot manage playing football, which bothers him a lot. He has remained at work, which consists primarily of office work. He now plans to study at the business school
Initially the pain slowly worsened and he became increasingly disabled due to pronounced pain on the backside of the right leg down to knee, periodically to heel level. He is currently only taking painkillers prior to physical activities
He has normal range of motion in the back although slightly reduced lateral flexion to the right, which causes known pain in the right buttock. Walking on heels and toes as well as one‐leg‐stand is normal and all reflexes can be triggered. There is normal strength and sensibility in the hip, knee, ankle and toes. MRI scan shows L5‐S1 prolapse on the right side with signs of nerve root pressure

Abbreviation: LBP, low back pain.

The questions, response options and classifications of the responses regarding work and activity were based on a previous study by Bishop, Foster, Thomas, and Hay (2008). The study by Bishop et al. did not investigate the domain of psychosocial risk factors, but as this is now considered essential in the management of LBP in the recent CPGs (Lin et al., 2020), the psychosocial domain was included in the current study.

### 2.6 Measuring adherence

The PTs' adherence to the CPGs was assessed on the basis of the advice they would give to the patients in the two vignettes on the three key guideline domains; (a) activity, (b) work and (c) psychosocial risk factors. Thus, for each vignette, there was one question for each guideline domain, with four possible answers for each question. For each question, one answer was classified as strictly in line with the CPGs, one was classified as partly in line with the CPGs and two were classified as not in line with the CPGs. The descriptions of all three‐guideline domains and possible answers are shown in Table 2.

**TABLE 2:** The PTs' response options regarding the three domains and the authors' classification of response

Key domains	Response options on advice	Classification of response
What is your advice when the patient asks what kind of activities he/she must perform:	1. Perform usual activities 2. Perform activities within the patient's tolerance 3. Perform only pain free activities 4. Limit all physical activities until pain disappears	Strictly in line Partly in line Not in line Not in line
What is your advice when the patient asks how to handle his/her work situation:	1. Return to normal work 2. Return to part time or light duties 3. Be off work until pain has improved 4. Be off work until pain has completely disappeared	Strictly in line Partly in line Not in line Not in line
To what extent do you assess the patient's psychological and social resources during your examination:	1. In the history‐taking, I ask about the patient's psychosocial condition/or use a questionnaire to screen for risk factors 2. I try to be aware of it, but I only have time to do a physical examination 3. I primarily relate to the possible tissue damage that underlies the patient's pain 4. I do not think I have the skills to assess the patient's psychosocial factors	Strictly in line Partly in line Not in line Not in line

### 2.7 The PTs' treatment modalities

In addition to answering the questions regarding advice on each of the three key domains, the PTs were asked to select which treatment modalities they would initiate in each vignette. A maximum of three treatment modalities out of 12 could be selected (see Appendix 1). The 12 treatment modalities were determined by the research team and validated by private and public PTs who, in the pilot test, were asked to add more treatment modalities if needed to make the responses as valid as possible to their daily practice. The treatment modalities were not included in the assessment of adherence to the three‐guideline domains, but were used for descriptive purposes only.

### 2.8 Data analysis

Data analysis was carried out using STATA 15 (Stata Corp, College Station, TX). Descriptive statistics (mean [*SD*] for continuous variables and frequencies [percentage] for categorical variables) were used to describe the PTs' characteristics as well as the adherence to the three domains and the treatment modalities. The difference in adherence to the three domains between the two groups (private vs. public PTs) was tested using the Chi‐squared test.

## 3 RESULTS

### 3.1 Participant flow

**FIGURE 1:**
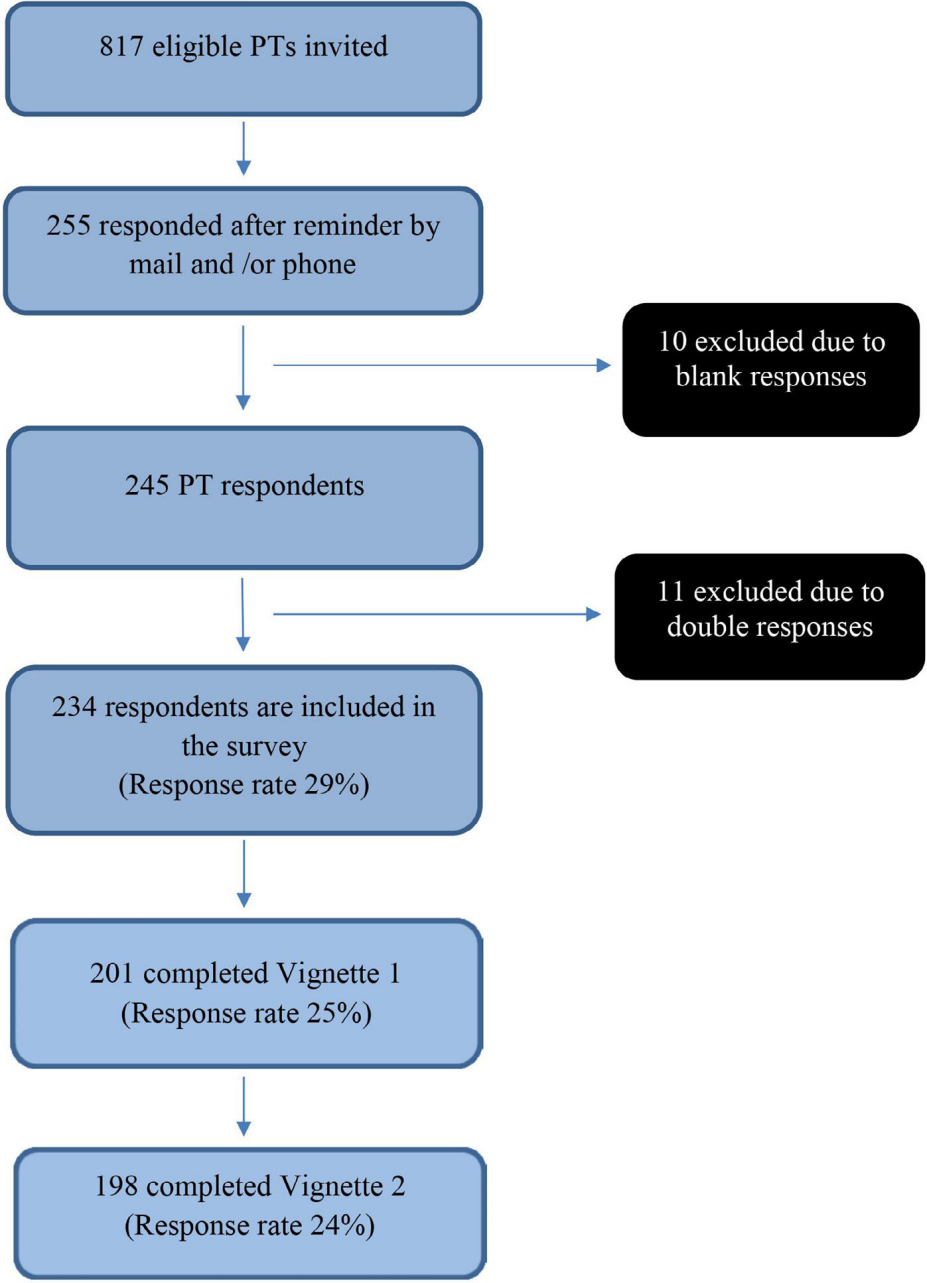
Flow chart of the PTs in the study. PT, physiotherapist

A total of 255 out of 817 invited PTs answered the electronic survey. Twenty‐one of the responses were removed, either due to blank responses (PTs who only opened the survey but did not respond, thereby being registered in REDCap as missing), or due to double responses (as it was not a unique link for the online survey, PTs who unintentionally answered the survey twice would appear as two different respondents in REDCap). Furthermore, several PTs did not complete the survey, resulting in a lower N in parts of the analysis. A total of 234 PTs were included in the study (response rate 29%). Participant flow is shown in Figure 1.

The characteristics of the participating PTs can be seen in Table 3. Overall, most were female (62%), and had more than 10 years of clinical experience (58%). In terms of differences between public and private PTs, there was more female in public PTs (80%) compared to private PTs (55%; *p* < .001). More public PTs (91%) allocated 60 min or more for the first consultation compared to private PTs (21%; *p* < .001). Private PTs treated more patients with LBP per week (79% treated six patients or more) compared to public PTs (59% treated six patients or more; *p* = .009).

**TABLE 3:** Characteristics of the participating PTs

Variables	PT (*N* = 234)	Private (*N* = 163)	Public (*N* = 71)
Sex*			
Male, *n* (%)	88 (38)	74 (45)	14 (20)
Age, mean (*SD*)	41.8 (10)	42.1 (10.5)	40.9 (9.0)
Clinical experience, *n* (%)			
<1 year	7 (3)	5 (3)	2 (3)
1–2 years	21 (9)	16 (10)	5 (8)
3–5 years	23 (10)	10 (6)	13 (20)
6–10 years	45 (20)	33 (21)	12 (18)
>10 years	130 (58)	96 (60)	34 (52)
Postgraduate training^a^, *n* (%)			
Yes	199 (88)	142 (89)	57 (86)
Number of LBP patients/week, *n* (%)*		(*N* = 160)	(*N* = 66)
<5/week	61 (27)	34 (21)	27 (41)
6–10/week	94 (42)	73 (46)	21 (32)
>10/week	71 (31)	53 (33)	18 (27)
≥60 min allocated to first consultation, *n* (%)*			
Yes	94 (42)	34 (21)	60 (91)

Abbreviation: LBP, low back pain.

^a^ Postgraduate training included courses (e.g., McKenzie, acupuncture) or continuing education (e.g., master's degree) within the management of patients with LBP after completion of their bachelor's degree in physiotherapy.

* Significant difference between private and public PTs, *p* < .005.

### 3.2 The PTs' adherence to the three key guideline domains

For Vignette 1, the proportion of PTs giving advice that was strictly in line with CPGs for the three domains was 32% (activity), 16% (work) and 82% (psychosocial factors). For Vignette 2, the corresponding results were 6% (activity), 53% (work) and 60% (psychosocial factors; Table 4). For the two domains ‘activity’ and ‘work’, there was no statistically significant difference between the adherence of private and public PTs, respectively. For the domain 'psychosocial risk factors' there was a divergence in adherence between the two groups in both Vignette 1 (92% vs. 77%, *p* = .03) and Vignette 2 (70% vs. 55%, *p* = .04) in favour of the public PTs, who adhered more strictly to the guideline domain.

**TABLE 4:** The PTs' advice regarding activity, work and assessing the. psychosocial risk factors

*Vignette 1*				
*Activity*	*PTs (N = 201)*	*Private (N = 140)*	*Public (N = 61)*	*p‐Value*
Strictly in line	64 (32)	48 (34)	16 (23)	.351
Partly in line	135 (67)	90 (64)	45 (74)	
Not in line	2 (1)	2 (1)	0 (0)	
Work				
Strictly in line	33 (16)	23 (16)	10 (16)	.383
Partly in line	159 (79)	110 (79)	49 (80)	
Not in line	9 (5)	7 (5)	2 (3)	
Psychosocial risk factors				
Strictly in line	164 (82)	108 (77)	56 (92)	.030
Partly in line	30 (15)	27 (19)	3 (5)	
Not in line	7 (3)	5 (4)	2 (3)	

*Vignette 2*				
*Activity*	*PTs (N = 198)*	*Private (N = 138)*	*Public (N = 60)*	*p‐Value*
Strictly in line	12(6)	9 (7)	3(5)	.863
Partly in line	180 (91)	124 (91)	56 (92)	
Not in line	6 (3)	4 (3)	2 (3)	
Work				
Strictly in line	105 (53)	69 (50)	36 (59)	.528
Partly in line	90 (45)	66 (48)	24 (39)	
Not in line	3 (2)	2 (2)	1 (2)	
Psychosocial risk factors				
Strictly in line	119 (60)	76 (55)	43 (70)	.035
Partly in line	33 (17)	29 (21)	4 (7)	
Not in line	46 (23)	32 (23)	14 (23)	

*Note*: Numbers in the table are *n* (%).

### 3.3 Post hoc analyses and results

Due to significant difference in terms of sex, the number of patients with LBP per week and the time allocated for first treatment between the private and public PTs, respectively, an exploratory post hoc analysis was performed using a chi‐squared test, to investigate whether guideline adherence was influenced by these factors.

In terms of adherence to the three‐guideline domains, no statistically significant difference was found between the subgroups, except for the domain 'psychosocial risk factors'. PTs who had allocated 60 min or more for the first consultation had a higher level of adherence compared with those with less than 60 min allocated (Vignette 1: *p* = .001; Vignette 2: *p* < .001; data not shown).

### 3.4 PTs' treatment modalities

The two most common treatment modalities were to instruct the patients in an exercise program (Vignette 1: 62%, Vignette 2: 70%) and to inform the patients about the benign nature and prognosis of LBP (Vignette 1: 66%, Vignette 2: 54%). The third most common treatment modality differed between the two vignettes. In Vignette 1, the PTs would examine the patient's worried thoughts about their LBP and in Vignette 2 they would teach the patient a better posture and provide ergonomic advice (data not shown).

## 4 DISCUSSION

The current study aimed to investigate the adherence by Danish PTs working in private clinics and public healthcare centres to CPGs for the management of LBP, specifically addressing the following three key guideline domains: (a) activity, (b) work and (c) psychosocial risk factors, and whether the adherence differed between private and public PTs. Additionally, the aim was to describe the PTs' treatment modalities for patients with LBP.

We found that the vast majority of the participating PTs partly adhered to two out of three domains and strictly adhered to only one out of the three‐guideline domains. More than 95% of the PTs would give advice regarding activity and work that was partly or strictly in line with the CPGs. In contrast, up to 23% did not adhere to the CPGs for assessing psychosocial risk factors in Vignette 2.This domain was also the only domain where there was a significant divergence in adherence between private and public PTs. Significantly more public PTs strictly adhered to CPGs for this domain. Most of the participating PTs also provided treatments recommended in the CPGs (information, exercises, identification of psychosocial issues and correction of working posture).

Assessment of guideline adherence is a complex matter, and although comparison of results across countries reveals great variation in guideline adherence, there are a number of possible reasons for the differences seen. Studies report difference in methods for measuring adherence (vignettes, bills of accounts, treatment options, etc.), difference in the definition of adherence, difference in the patient described in the vignette (symptom complaints, personal characteristics) as well as difference in the key domains measured (activity, work, reference to specialist and X‐ray, medicine, bed rest etc.; Casserley‐Feeney, Bury, Daly, & Hurley, 2008; Fritz et al., 2007; Hendrick et al., 2013; Keating et al., 2016; Ladeira et al., 2015; Learman et al., 2014). These variations of assessing guideline adherence may explain the differences in results between studies described below.

### 4.1 Key guideline domain: Activity

In the current study, none of the vignettes included patients with symptoms suggesting serious spinal pathology. Still, the PTs were cautious about advising the patients to ‘perform usual activities’. The recommendation in the CPGs regarding activity may be considered equivocal, because it states that advising patients to ‘stay active within pain tolerance’ might induce fear‐avoidance beliefs, thereby contributing to a reduced level of activity (Danish Health Authority, 2016a). On the other hand, the CPGs emphasize that patients should be encouraged to gradually increase their level of activity. PTs may therefore be confused about which advice they should give their patients (perform usual activities or perform activities within pain tolerance).

The results are comparable to those reported in studies by Hendrick et al. and Keating et al., showing the majority of PTs provided advice that was strictly or partly in line with the CPGs. This is in opposition to Souza et al., who showed that less than 20% of the PTs advised their patients to pursue or maintain an active lifestyle despite similar methods for measuring adherence (de Souza et al., 2017; Hendrick et al., 2013; Keating et al., 2016). Despite the Danish PTs advising patients to remain active, there is still room for improvement.

### 4.2 Key guideline domain: Work

The PTs' advice regarding work differed between the two vignettes. The majority of PTs would advise the patient in Vignette 1 (on sick leave from her work as a healthcare assistant) to ‘return to part time or light duties’ at work. It could be considered whether this advice should have been categorized as strictly in line as the CPGs recommend that giving advice regarding temporary changes in work functions or working hours is also an option in line with best evidence. However, due to the fact that the CPGs emphasize that staying at work most often is beneficial for maintaining function, and since the patient did not show any signs of red flags (serious spinal pathology) or radiculopathy, ‘return to normal work’ was therefore considered to be strictly in line with CPGs. The majority of the PTs would advise the patient in Vignette 2 (not on sick leave from his office work) to stay at work full‐time, but 45% still would advise him to reduce his work hours. The results for the work domain in our study are comparable to those from the study by Hendrick et al., who showed that the majority of the PTs in New Zealand gave advice regarding work that was partly in line with the guidelines (Hendrick et al., 2013). However, it is not clear why the PTs in the current study were cautious in their advice regarding work in Vignette 2. The result shows that there still is room for improvement in terms of higher adherence to CPGs by the Danish PTs on the work domain.

### 4.3 Key guideline domain: Psychosocial risk factor

With regard to the psychosocial domain, the PTs adhered differently in the two vignettes, with 23% of public PTs reporting that they would not assess psychosocial risk factors in Vignette 2. The CPGs recommend screening *all* patients for psychosocial risk factors (Danish Health Authority, 2013, 2016a, 2017), which is supported by a recent review by Alhowimel et al., underlining the importance of assessing the patients' psychosocial risk factors due to the association between baseline psychosocial factors and post‐treatment disability and pain (Alhowimel, AlOtaibi, Radford, & Coulson, 2018). The authors were unable to find any literature assessing PTs' adherence to the domain ‘psychosocial risk factors’, despite clear evidence of the negative impact of these factors on prognosis and responsiveness to LBP management (Alhowimel et al., 2018; Koes et al., 2010; Pincus, Burton, Vogel, & Field, 2002). However several other studies have shown that it may be challenging to allocate the amount of time that is required for integration of psychosocial risk factor assessment into clinical practice, and further, that PTs lack knowledge and skills to manage these factors (Cowell et al., 2018; Driver, Kean, Oprescu, & Lovell, 2017; Foster & Delitto, 2011; Synnott et al., 2015). The result confirms the importance of improving the implementation of assessment and management of psychosocial risk factors in PT practice.

### 4.4 Private versus public PTs

In the current study, the main difference found between private and public PTs was in terms of adherence to the psychosocial domain. The difference in treatment needs in public and private PT practice, as described in the Setting section, may explain why the private PTs might have more biomedical focus in their assessment and management. However, it is recommended in the CPGs to identify all patients' psychosocial risk factors as soon as possible, and addressed through treatment if necessary, and therefore both groups should adhere to the key domain independent on the patients treatment needs. The result may likewise have been due to the public PTs reporting that they allocated more time to the first consultation (91% of public vs. 22% of private PTs allocating 60 min or more). The difference in time spent on the first consultation is probably explained by the different payment/reimbursement systems for the two groups. In Denmark, private PTs receive payment for the number of patients, whereas public PTs get a monthly salary independent of the number of patients they see. This is consistent with a study by Casserley‐Feeney et al., who found that PTs working in the municipalities spent more time on consultations than PTs working in private clinics (Casserley‐Feeney et al., 2008). The result shows that more efforts must be made by the private PTs compared to public PTs in terms of implementation of the psychosocial domain.

### 4.5 Treatment modalities

The majority of the participating PTs provided treatments which are recommended in the CPGs. Only the third most common treatment modality in Vignette 2 (posture and ergonomic advice) is not mentioned in any of the CPGs. This treatment modality might be explained by PTs having a biomedical focus (Nijs, Roussel, Paul van Wilgen, Köke, & Smeets, 2013). However, only a very small percentage (1–3%) of PTs would provide passive treatments such as cold and heat treatment, electrotherapy or acupuncture, compared with the Danish study from 2003 which reported an over‐use of passive treatments (17–32% depending on the type of treatment; Hamm et al., 2003). A study undertaken by Bernhardsson et al. also found that less than 5% of Swedish PTs reported use of electrotherapy as a treatment modality (Bernhardsson, Öberg, Johansson, Nilsen, & Larsson, 2015). The decrease in the use of electrotherapy may be due to recent findings demonstrating a lack of evidence for its effect.

### 4.6 Strengths and limitations

To the best of our knowledge, this study is the first Danish cross‐sectional study to have assessed adherence to CPGs for LBP by Danish PTs working in community‐based settings over the past 10 years, during which period several CPGs have been published in the field of LBP. The strengths of the current study are the thorough development of the questionnaire based on an exhaustive survey of all national CPGs and recommendations, pilot‐testing and quality assurance of the survey and the vignettes by clinical experts in the field. Both private and public PTs were involved in the development and pilot‐testing of the survey, and the PTs were furthermore invited to a discussion following the survey, where the results of the survey were reflected on, and barriers for guideline adherence were discussed.

The low response rate of 29% might be partly due to the length of the survey, estimated to be approximately 20 min, which they were notified of in the participant information letter. Only 22% of the invited private PTs responded to the survey, whereas around 85% of the public PTs responded, indicating that the findings are not representative of private PTs working with LBP care in Denmark. Most likely, the results provide an optimistic view of the PTs' adherence to CPGs, as those responding to the survey were probably those who were engaged in professional development. Low response rates are, however, not uncommon for surveys among health care professionals working in private clinics, and other studies have reported similar low response rates of between 14.5 and 36% (de Souza et al., 2017; Hendrick et al., 2013; Keating et al., 2016; Ladeira et al., 2015).

Another limitation of this study is the lack of consensus on the definition of adherence as well as lack of standardized instruments for measuring adherence, indicating the complexity of the phenomenon. In the current study, the classification of responses for the two domains of activity and work were based on a previous study by Bishop et al. (2008), allowing for comparability of the results. Although vignettes are generally accepted as a valid measure of adherence, one study has shown a poor correlation between the PTs' response to the vignettes and what blinded patients described, to have been advised about activity and work after visiting the PT (Brunner, Probst, Meichtry, Luomajoki, & Dankaerts, 2016). A more valid instrument would be desirable. However, due to lack of time and resources, vignettes were considered the best option.

## 5 CONCLUSION

Overall, the participating Danish PTs partly adhered to two out of three‐guideline domains, and would offer treatment modalities in line with CPGs. However, strict adherence was seen in only one out of the three key domains. The majority of the PTs assessed the patients' psychosocial risk factors, but did not consider screening for psychosocial risk factors as a standard part of the assessment. Also, the PTs were cautious to advise the patients to return to usual activities and work. Public PTs were more likely to be strictly in line with the CPGs for assessing the psychosocial risk factors.

## AUTHOR CONTRIBUTIONS

All authors have contributed to the conception, design, analyses and interpretation of the data and been involved in drafting or revising the manuscript.

## ETHICAL APPROVAL

The study is registered in ClinicalTrials.gov (Reg. No. NCT03426410). Under the Danish Act concerning Research Ethics Review of Health Research Projects, health research projects using surveys and questionnaires do not need ethics approval (http://en.nvk.dk/rules‐and‐guidelines/act‐on‐research‐ethics‐review‐of‐health‐research‐projects). The data collection was approved by the Danish Data Protection Agency (Case No. 1‐16‐02‐863‐17) and consent from the physiotherapists was assumed if they responded to the survey.

## IMPLICATIONS FOR PHYSIOTHERAPY PRACTICE

This study shows that there is room for improvement concerning PTs' adherence with CPGs in the field of LBP. It is therefore of great importance to focus on enhancing the implementation of the existing CPG recommendations for managing patients with LBP. Time allocated to the first treatment can be of importance in order to assess and address the patients' psychosocial risk factors. However, this needs to be supported by future research.

## Appendix 1 1

**Table 5:** The 12 possible treatment modalities for the two vignettes

A) Teach her/him a better posture and ergonomics
B) Instruct her/him to use heat / cold to relieve pain
C) Examine any worried thoughts she/he has about her/his low back pain
D) Instruct her/him in mindfulness exercises
E) Explain that a rupture has occurred in the disc, so the disc core presses a nerve
F) Instruct her/him in a training program / offer to participate in a team
E) Inform her/him about the benign nature and prognosis of low back pain
G) Give her/him direction‐specific exercises (McKenzie)
H) Use manual techniques
I) Give advice about pain management and / or refer to a pain management course
J) Apply electrical therapy (eg. ultrasound, TENS, other)
K) Apply acupuncture
